# Microbiota and Metatranscriptome Changes Accompanying the Onset of Gingivitis

**DOI:** 10.1128/mBio.00575-18

**Published:** 2018-04-17

**Authors:** Emily M. Nowicki, Raghav Shroff, Jacqueline A. Singleton, Diane E. Renaud, Debra Wallace, Julie Drury, Jolene Zirnheld, Brock Colleti, Andrew D. Ellington, Richard J. Lamont, David A. Scott, Marvin Whiteley

**Affiliations:** a John Ring LaMontagne Center for Infectious Disease, The University of Texas at Austin, Austin, Texas, USA; b Department of Molecular Biosciences, The University of Texas at Austin, Austin, Texas, USA; c Center for Systems and Synthetic Biology, College of Natural Sciences, The University of Texas at Austin, Austin, Texas, USA; d School of Dentistry, University of Louisville, Louisville, Kentucky, USA; e School of Biological Sciences, Georgia Institute of Technology, Atlanta, Georgia, USA; f Emory-Children’s Cystic Fibrosis Center, Atlanta, Georgia, USA; UT Southwestern Medical Center Dallas

**Keywords:** dysbiosis, gingivitis, metatranscriptome, oral microbiology, periodontitis

## Abstract

Over half of adults experience gingivitis, a mild yet treatable form of periodontal disease caused by the overgrowth of oral microbes. Left untreated, gingivitis can progress to a more severe and irreversible disease, most commonly chronic periodontitis. While periodontal diseases are associated with a shift in the oral microbiota composition, it remains unclear how this shift impacts microbiota function early in disease progression. Here, we analyzed the transition from health to gingivitis through both 16S v4-v5 rRNA amplicon and metatranscriptome sequencing of subgingival plaque samples from individuals undergoing an experimental gingivitis treatment. Beta-diversity analysis of 16S rRNA reveals that samples cluster based on disease severity and patient but not by oral hygiene status. Significant shifts in the abundance of several genera occurred during disease transition, suggesting a dysbiosis due to development of gingivitis. Comparing taxonomic abundance with transcriptomic activity revealed concordance of bacterial diversity composition between the two quantification assays in samples originating from both healthy and diseased teeth. Metatranscriptome sequencing analysis indicates that during the early stages of transition to gingivitis, a number of virulence-related transcripts were significantly differentially expressed in individual and across pooled patient samples. Upregulated genes include those involved in proteolytic and nucleolytic processes, while expression levels of those involved in surface structure assembly and other general virulence functions leading to colonization or adaptation within the host are more dynamic. These findings help characterize the transition from health to periodontal disease and identify genes associated with early disease.

## IMPORTANCE

Although more than 50% of adults have some form of periodontal disease, there remains a significant gap in our understanding of its underlying cause. We initiated this study in order to better characterize the progression from oral health to disease. We first analyzed changes in the abundances of specific microorganisms in dental plaque collected from teeth during health and gingivitis, the mildest form of periodontal disease. We found that the clinical score of disease and patient from whom the sample originated but not tooth brushing are significantly correlated with microbial community composition. While a number of virulence-related gene transcripts are differentially expressed in gingivitis samples relative to health, not all are increased, suggesting that the overall activity of the microbiota is dynamic during disease transition. Better understanding of which microbes are present and their function during early periodontal disease can potentially lead to more targeted prophylactic approaches to prevent disease progression.

## INTRODUCTION

**FIG 1:**
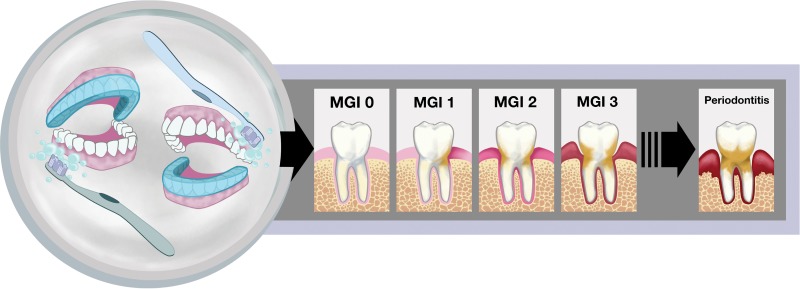
Study design and visualization of the progression from health to periodontal disease. On the left, the covered or uncovered teeth depict the study design utilized, in which an acrylic stent (shown in blue) was worn to cover either the entire top or bottom set of teeth during brushing throughout the course of the experiment. The images on the right illustrate the clinical symptoms associated with gingivitis that were scored by trained dental professionals in this study. An MGI score of 0 represents a healthy tooth with no indication of inflammation (shown by increasing redness at the gum) or plaque (tan color on tooth). Healthy periodontia progress through various degrees of gingivitis as depicted by the MGI 1, MGI 2, and MGI 3 panels and can eventually progress to the destructive gum disease, chronic periodontitis, shown on the far right.

Oral microbes are found as an organized and complex polymicrobial biofilm community potentially containing at least 750 unique bacterial species with various genetic potentials (1–5). While these microbes normally coexist within the mouth as commensals, infrequent or inadequate cleaning can lead to periodontal disease in a susceptible host (5, 6). The mildest form of periodontal disease, gingivitis, is characterized by plaque buildup in the subgingival crevice of teeth (7) and inflammation of the gums (8, 9). Gingivitis symptoms can be eliminated and the gums restored to a healthy state through professional dental cleaning. Untreated gingivitis, however, can progress to chronic periodontitis (10), an irreversible periodontal disease characterized by chronic inflammation, destruction of gum tissue, and ultimately loss of both tooth attachment and alveolar bone (9, 11) (Fig. 1).

A number of studies have harnessed the power of next-generation sequencing technology to characterize both the composition and function of the oral microbiota during health or periodontitis (12–21). A distinct phylogenetic structure in health relative to periodontitis has been revealed through 16S rRNA gene and shotgun metagenomic sequencing (12, 17, 19). Other studies have performed metagenome (13, 14, 18, 20) or metatranscriptome (15, 16, 21, 22) analyses to examine the functional potential of the oral microbiota in health and disease. Several of these studies have revealed the presence and expression patterns of genes associated with pathogenesis during periodontal disease. Although the genomes of many commensals found during periodontal health contain virulence-related genes, many of these genes are either uniquely present or more highly expressed in samples collected from patients with chronic periodontitis (14). Additionally, several studies have revealed that gene expression patterns of the oral microbiota are patient specific despite matched patient health status, suggesting that factors unique to each individual can shape microbial community structure and activity (14, 16). These studies have collectively expanded our understanding of how both community structure and function differ between periodontitis and health as well as across individuals.

Several other studies have focused on understanding changes in microbial community structure during health relative to early periodontal disease, gingivitis (6, 8, 9, 23). For example, Huang et al. reported that unique taxonomic groups are found in plaque samples from either healthy patients or those with gingivitis (23). In another study, 50 adults with naturally occurring gingivitis were restored to “baseline” dental health and then subjected to a 3-week experimental gingivitis treatment. Researchers found that 27 bacterial genera were differentially distributed between baseline and gingivitis, with 5 of these showing elevated abundance in health and 22 having elevated abundance in gingivitis (8). While these studies have helped to elucidate the community-level shifts that occur during the transition from health to gingivitis, the functional changes that occur during the progression of periodontal disease have not yet been examined.

Understanding the functional contributions of the oral microbiota as periodontal disease develops is critical in order to develop more effective prophylactic treatments for preventing this all-too-common disease. The work here assesses both community and functional changes of the human oral microbiota during the transition from health to an inflammatory periodontal disease. Analysis of subgingival plaque from a 3-week experimental gingivitis treatment cohort revealed that similarity of microbiota compositions between samples is significantly correlated with both clinical severity of gingivitis and patient but not oral hygiene status. The relative abundances of seven of the most highly represented genera were found to differ significantly between patient-matched samples from teeth at different stages of gingivitis. With few exceptions, relative genus abundance as determined by 16S rRNA sequencing and relative transcript abundance determined by metatranscriptome sequencing (RNA-seq) were in agreement. Metatranscriptome sequencing of the plaque samples revealed that many genes significantly differentially expressed during gingivitis relative to health have virulence-related functions and that while many of these functions serve to potentially promote tissue destruction and disease, the role of other virulence-related gene products during early stages of disease is less clear. These data provide the characterization of changes in microbial activity that occur during the early stages of periodontal disease, which can potentially serve as targets to prevent further disease progression.

## RESULTS

### Microbiota composition is correlated with clinical gingivitis index and patient.

Subgingival plaque samples pooled from two brushed or unbrushed teeth in 10 individuals were collected at three different time points (as described in Materials and Methods) and were analyzed based on clinical disease parameters and community composition. Table S1 in the supplemental material shows the day after study onset on which the sample was collected as well as two clinical measurements: the modified gingival index (MGI) score, or clinical index of gingivitis severity of sampled teeth (depicted in Fig. 1), and probing depth (PD), which measures the severity of tooth-gum attachment loss associated with periodontal diseases (24, 25). While MGI score increased over time in all teeth regardless of oral hygiene status (i.e., brushed or unbrushed), the magnitude of this increase was patient specific. Although there were, on average, higher MGI scores for unbrushed teeth at each visit than for brushed teeth, this difference was not statistically significant (Table S1). Similarly, no significant difference in PD was found between brushed and unbrushed teeth between the start (visit 2) and end (visit 6) of the study (Table S1).

10.1128/mBio.00575-18.7 TABLE S1  Patient clinical plaque sample data. Patient number and demographics, oral hygiene status (brushed or not brushed) of teeth, modified gingival index (MGI) score, and probing depth (PD; millimeters) are indicated. MGI scores progressed from healthy (0) to mild gingivitis (2)—see Materials and Methods (and Fig. 1) for an expanded definition of MGI score. MGI and PD measurements represent the average from 2 sampled teeth for each condition. The average measurements and standard errors of the means for brushed and unbrushed samples across all patients at each visit are indicated. Student’s *t* test revealed no significant differences in MGI score and PD between brushed and unbrushed teeth. Download TABLE S1, TIF file, 1.6 MB. Copyright © 2018 Nowicki et al. 2018 Nowicki et al. This content is distributed under the terms of the Creative Commons Attribution 4.0 International license.

We first assessed the microbial composition and diversity within the collected plaque samples through 16S rRNA sequencing, allowing us to focus on organisms actively producing protein and thus with the greatest potential to influence community activity (26). We performed alpha (within-sample)-diversity analysis of sequenced 16S rRNA reads from subgingival plaque samples obtained from either brushed or unbrushed teeth. This revealed no statistically significant difference in Shannon index, which measures both species evenness and abundance, between samples originating from brushed and from unbrushed teeth (Fig. S1A). In contrast, samples grouped by clinical index of patient gingivitis severity (MGI score) showed significant differences in alpha-diversity in both the high- and medium-MGI samples compared to the low-MGI group (Fig. S1B, *P* < 0.005). While Shannon indexes did not differ among most of the samples grouped by patient, samples collected from patients 6 and 14 did have higher diversity (Fig. S1C).

10.1128/mBio.00575-18.1 FIG S1  Shannon index showing within-sample diversity (alpha-diversity) of 16S rRNA sequencing reads from all samples. Samples are grouped by oral hygiene status of the tooth (i.e., whether or not tooth was brushed) (A), MGI score (low MGI = 0 to 0.5, medium MGI = 1 to 1.5, high MGI = 2; both medium and high MGI were significantly higher than low MGI, *P* < 0.005) (B), and patient (C). Error bars show standard deviations in samples. Download FIG S1, TIF file, 1.2 MB. Copyright © 2018 Nowicki et al. 2018 Nowicki et al. This content is distributed under the terms of the Creative Commons Attribution 4.0 International license.

**FIG 2:**
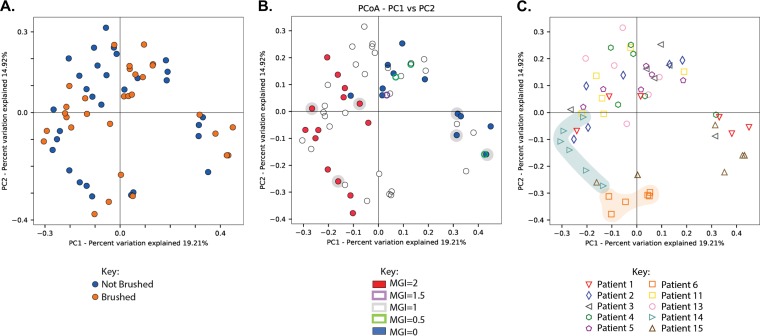
PCoA analysis of subgingival plaque sample 16S rRNA sequencing reads. 16S rRNA sequencing reads were categorized into distinct operational taxonomical units (OTUs) by mapping to the HOMD (v14.51) reference database using standard QIIME scripts. Beta-diversity between samples was measured through a Bray-Curtis dissimilarity analysis, and the principal coordinates (PCo) are plotted and colored by brushed/not-brushed plaque samples (*P* = 0.68) (A), MGI score (*P* = 0.001) (samples chosen for RNA-seq are highlighted) (B), and patient of origin (highly clustered patients are highlighted) (*P* = 0.001) (C).

Beta (between-sample)-diversity was also assessed via a Bray-Curtis dissimilarity analysis of 16S rRNA reads and visualized using principal-coordinate analysis (PCoA). Surprisingly, this analysis revealed no significant differences between samples collected from teeth with different oral hygiene statuses, suggesting that in our study brushing had no meaningful impact on the composition of the subgingival plaque microbiota (Fig. 2A) (*P* = 0.685). Similarly, no significant phylogenetic similarity was found between samples when grouped by the net changes in proinflammatory cytokine interleukin-8 (IL-8), MMP-8, or MMP-9 (Fig. S3A, B, and C) between day 1 and day 21 of the experimental gingivitis study. Importantly, beta-diversity analysis revealed significantly different clustering of all collected samples by MGI score (Fig. 2B) (*P* = 0.001). This clustering was most distinct between samples collected from teeth with the lowest and highest MGI scores in the data set (MGI = 0 and MGI = 2). Significant or marginally significant clustering by MGI score was also found when controlling for visit at which the sample was collected (Fig. S2) (visit 2, *P* = 0.025; visit 3, *P* = 0.012; visit 6, *P* = 0.08) and for patient (Fig. 2C) (permutational multivariate analysis of variance [PERMANOVA], *P* = 0.001), though some study subjects did display tighter clustering than others (i.e., patients 6 and 14). Together, these data suggest that the strongest predictors of microbiota phylogenetic similarity between samples are the clinical index of disease severity (MGI score) and the patient from whom the sample originated.

10.1128/mBio.00575-18.2 FIG S2  PCoA clustering of 16S rRNA sequencing samples by MGI score and visit. 16S rRNA sequencing reads were categorized into distinct operational taxonomical units (OTUs) by mapping to the HOMD (v14.51) reference database using standard QIIME scripts. Beta-diversity between samples was measured through a Bray-Curtis dissimilarity analysis of the different OTUs within samples. Coloring indicates severity of gingivitis by MGI score, while the shape of each point indicates the day on which the sample was collected for visit 2 (MGI, *P* = 0.025) (A), visit 3 (MGI, *P* = 0.012; time point, *P* = 0.007) (B), and visit 6 (MGI, *P* = 0.08; time point, *P* = 0.122) (C). Download FIG S2, TIF file, 1.1 MB. Copyright © 2018 Nowicki et al. 2018 Nowicki et al. This content is distributed under the terms of the Creative Commons Attribution 4.0 International license.

10.1128/mBio.00575-18.3 FIG S3  PCoA clustering of 16S rRNA sequencing samples by changes in cytokine levels. 16S rRNA sequencing reads were categorized into distinct operational taxonomical units (OTUs) by mapping to the HOMD (v14.51) reference database using standard QIIME scripts. Beta-diversity between samples was measured through a Bray-Curtis dissimilarity analysis of the different OTUs within samples, and the principal coordinates were plotted and colored by net fold change in IL-8 concentration during study (*P* = 0.347) (A), net fold change in MMP-8 concentration during study (*P* = 0.831) (B), and net fold change in MMP-9 concentration during study (*P* = 0.067) (C). Download FIG S3, TIF file, 1.3 MB. Copyright © 2018 Nowicki et al. 2018 Nowicki et al. This content is distributed under the terms of the Creative Commons Attribution 4.0 International license.

### Shifts in relative genus abundance occur during the transition from health to gingivitis.

**FIG 3:**
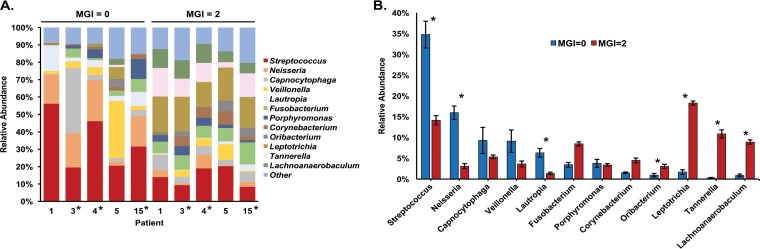
Composition of microbial communities from dental plaque samples assessed by 16S rRNA sequencing analysis. (A) Relative abundance of genera in the oral subgingival plaque community representing ≥1% within each subgingival plaque sample based on 16S rRNA sequencing. Samples further analyzed via RNA-seq metatranscriptome analysis are indicated with an asterisk. (B) Changes in average genus percent abundance across 5 patients (patients 1, 3, 4, 5, and 15) from samples collected from teeth with an MGI score of 0 (clinical health) or an MGI score of 2 (clinical disease). Error bars represent standard error. Genera that change significantly from MGI = 0 to MGI = 2 samples are indicated with an asterisk (*P* < 0.05).

In light of our beta-diversity results, patient-matched samples collected from teeth with the lowest MGI score (MGI = 0; clinically healthy) or highest MGI score (MGI = 2; clinical gingivitis) were assessed to determine the changes in relative abundance of microbial genera during disease progression. Patients 1, 3, 4, 5, and 15 had plaque samples collected from teeth that met this criterion (i.e., teeth with an MGI score of 0 and an MGI score of 2) and thus were included in this analysis; patient samples collected from teeth that did not have an MGI score of 0 were excluded from this analysis. Samples collected from teeth with an MGI score of 0 at visit 3 were selected for further analysis due to increased amounts of nucleic acids in these samples relative to those from visit 2. Since our beta-diversity analysis revealed no significant effect of brushing on sample clustering, sample data from both brushed and unbrushed teeth, both with an MGI score of 0, were averaged when possible. Operational taxonomic units (OTUs) with low (<1%) relative abundance were filtered from the samples analyzed, and the remaining OTUs were pooled by genus. Clear shifts in the relative abundance of specific genera between health and gingivitis occurred within each patient analyzed (Fig. 3A).

The mean count of genera in samples collected from teeth at times of health and gingivitis in these same five patients analyzed in Fig. 3A was analyzed for statistically significant changes using a Kruskal-Wallis nonparametric analysis of variance (ANOVA) test, revealing 7 bacterial genera with significantly different abundance between samples at different stages of gingivitis in the same patients. Of these, *Streptococcus*, *Neisseria*, and *Lautropia* had significantly higher relative abundance in samples collected from healthy teeth (MGI = 0, visit 3), while *Oribacterium*, *Leptotrichia*, *Tannerella*, and *Lachnoanaerobaculum* were significantly more abundant during gingivitis (Fig. 3B). This suggests that a potential dysbiosis of microbial composition and increased abundance of periodontal pathogens occur during the early transitional disease state.

### Overall RNA-seq transcript data correlate with 16S rRNA sequencing data.

We next wanted to elucidate changes in the specific activities of the subgingival plaque communities during disease progression. As a proxy for functional activity, we used a metatranscriptome sequencing approach (RNA-seq) to compare gene expression changes in plaque samples collected from teeth during a state of clinical gingivitis relative to health (samples with an MGI of 2 versus those with an MGI of 0). Of the 5 study participants (patients 1, 3, 4, 5, and 15) from whom samples were obtained from teeth with an MGI score of 0 at visit 3 and an MGI score of 2 at visit 6, patients 3, 4, and 15 were selected for RNA sequencing. These three samples were chosen because they had higher RNA concentrations and quality scores than samples from patients 1 and 5. Thus, we compared gene expression in samples collected from teeth during health (MGI = 0) and gingivitis (MGI = 2) that were both patient and time point (visit) matched. The total number of reads obtained after trimming and read mapping statistics and average coverage for each sample are shown in Fig. S4C. Given the number of samples, the minimum average sequencing depth, and the effect size fold cutoff of 2.75, we calculated our statistical power to be greater than 0.8.

10.1128/mBio.00575-18.4 FIG S4  Simulated mapping analysis and mapping statistics. (A and B) One million single-end simulated reads were generated through wgsim from either the entire metatranscriptome (A) or a subset of genes with EC annotations (B). Mapping was performed using Bowtie 2 with parameters used in the study to the entire transcriptome in both cases. Correct reads were identified by right mapping to the gene in which the simulated read originated. For species-level simulation, increasing the read length from the 30 bp used in the analysis would only marginally increase reads for downstream analysis. For EC-level simulation, we achieve >80% correct mapping with read lengths of 18 bp. (C) Table showing read mapping statistics for each sample. Download FIG S4, TIF file, 1.9 MB. Copyright © 2018 Nowicki et al. 2018 Nowicki et al. This content is distributed under the terms of the Creative Commons Attribution 4.0 International license.

**FIG 4:**
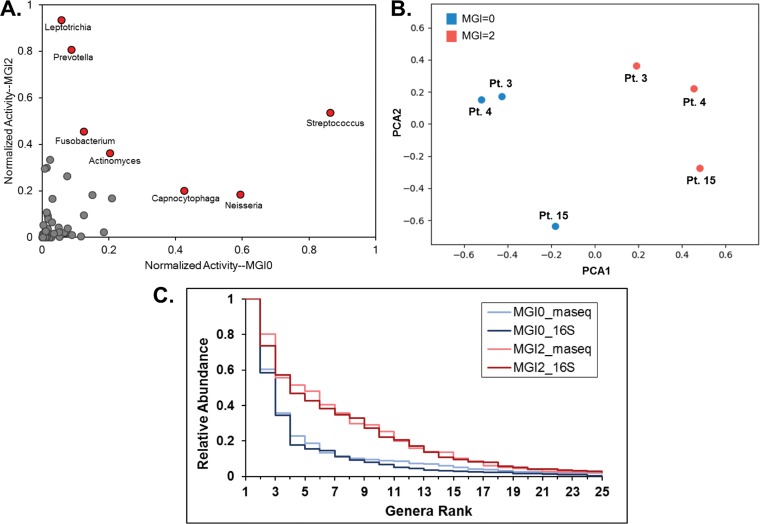
Comparison of transcriptomic data between disease states, patients, and taxonomic abundance. (A) Transcriptomic activity is normalized within sample to the highest-activity genus and aggregated by genus across the three samples for MGI0 samples (*x* axis) and MGI2 samples (*y* axis), allowing a comparison of relative sample abundance between teeth at states of clinical health (MGI0) and clinical disease (MGI2). Red data points indicate genera with the highest relative abundance in either MGI = 0 or MGI = 2 samples. (B) PCA of RNA-seq reads originating from each patient sample colored by MGI score. (C) Rank abundance curves are shown for MGI = 0 and MGI = 2 samples for both taxonomic and transcriptomic analyses.

We first compared bacterial activities from transcriptomic data by plotting the within-sample normalized activities from both disease types after aggregating counts to common genera (Fig. 4A). The most abundant genera in averaged plaque samples at MGI = 0 or MGI = 2 were identified and are indicated in Fig. 4A by red points. In health, these genera were found to be *Streptococcus*, *Neisseria*, and *Capnocytophaga*, with the former two having been identified in the 16S taxonomic analysis. We observed the genera favoring disease to be *Leptotrichia* (observed in 16S analysis) and *Prevotella* and also *Fusobacterium*, for which metatranscriptomic influence in promoting gingivitis has been previously reported (27). In addition to looking at changes in transcriptomic activity or read counts, which essentially measure the overall abundance of each genus in disease compared to health, we also analyzed the fold change of each genus during progression to gingivitis. While fold change is more sensitive to lower-expression genera, we can use this metric to characterize the largest relative community changes. We identified the most discordant genera in our transcriptomic analysis in samples collected during gingivitis as *Tannerella*, *Treponema*, and *Leptotrichia*, while *Haemophilus*, *Granulicatella*, and *Neisseria* were most discordant in samples collected during clinical health (Fig. S5A). We next employed principal-component analysis (PCA) to dissect the sample-level trends and observed well-defined clustering of the MGI = 0 and MGI = 2 patient samples (Fig. 4B), thus showing distinct transcriptomic differences between samples during health and disease.

10.1128/mBio.00575-18.5 FIG S5  Genus- and species-level comparison for 16S rRNA and RNA sequencing data. (A) Genus comparison using log_2_ fold change from patient-matched healthy and periodontitis samples in both 16S rRNA (red) and RNA-seq (blue) analyses. (B and C) Taxonomic abundances from MGI = 0 and MGI = 2 samples (B) and transcriptomic abundances from MGI = 0 and MGI = 2 samples (C). An asterisk indicates a significant difference (*P* < 0.05). Reads were mapped to genes and aggregated based upon species of origin. Relative abundance was calculated by dividing each species count by the highest-count species within each sample and averaged across all three samples. Download FIG S5, TIF file, 1.9 MB. Copyright © 2018 Nowicki et al. 2018 Nowicki et al. This content is distributed under the terms of the Creative Commons Attribution 4.0 International license.

We next wanted to analyze the relationship between the taxonomic abundances and transcriptomic activity in samples collected from the same teeth during health or disease. To achieve this, we generated a rank-abundance plot to compare the bacterial community diversities (Fig. 4C). Briefly, the counts of each genus were normalized by dividing by the most abundant/active genus within each sample and ordering by rank. We observe that, after averaging across three patients for each disease state (MGI = 0 versus MGI = 2) and sequencing type, we found that samples collected during gingivitis exhibit greater bacterial diversity than those from healthy patients, and this trend is preserved through both the 16S and transcriptomic analyses. To more directly compare specific genera between abundance and activity, we performed regression analysis of average normalized genera in both MGI = 0 and MGI = 2 samples of the 25 most abundant genera (Fig. S6A and B). We find that microbial genus activity and abundance in samples collected from teeth at a clinically healthy disease state are more closely correlated than in samples collected from teeth at a clinically diseased state. When analyzing the fold change of genera in healthy and disease samples, we find similar trends in the fold changes of each genus from disease to health in both the taxonomic and transcriptomic data (Fig. S5A). Altogether, our data show that while the total abundance of certain genera differs when analyzed by either 16S rRNA sequencing or metatranscriptome sequencing, the overall trends in abundance fold change and rank demonstrate high concordance between the two data sets.

10.1128/mBio.00575-18.6 FIG S6  Genus abundance comparison between 16S rRNA and RNA-seq data. (A) Average relative abundances of the most abundant genera in MGI = 0 samples are plotted for both 16S rRNA and RNA-seq data. (B) Average relative abundances of the most abundant genera in MGI = 2 samples are plotted for both 16S rRNA and RNA-seq data. Download FIG S6, TIF file, 1.1 MB. Copyright © 2018 Nowicki et al. 2018 Nowicki et al. This content is distributed under the terms of the Creative Commons Attribution 4.0 International license.

### Virulence-related gene expression is elevated during the transition from oral health to gingivitis.

We began our functional analysis by pooling read counts across all transcriptomes present in our samples by combining counts for transcripts with the same Enzyme Commission (EC) number. By pooling gene expression data for gene products involved in the same biochemical reaction, we were able to assess the overall activity of the microbial community. Further, previous work from our laboratory has shown that the expression of genes pooled by common metabolic function (EC number) is considerably less variable than the expression of individual organismal genes (16). Differential gene expression between samples collected from the same teeth during health (MGI = 0) and gingivitis (MGI = 2) was analyzed using the R package DESeq2 (27). Of the 2,241 unique EC numbers analyzed (representing 111,778 of the 625,371 total unique open reading frames [ORFs] with reads mapping to our reference data set), 191 (8.5%) enzymes were significantly (*P* ≤ 0.05) upregulated by 2.75-fold or greater in gingivitis relative to health while 180 (8.0%) were significantly downregulated (Data Set S1). Overall, our data show that metabolic pathways more strongly associated with health (downregulated during disease) include genes involved in ascorbate and aldarate metabolism, porphyrin and chlorophyll metabolism, carbon fixation in prokaryotes, the pentose phosphate pathway, antibiotic biosynthesis, and pyruvate metabolism. Metabolic pathways more strongly associated with disease (upregulated during disease) include genes involved in pyrimidine metabolism, vitamin B_6_ metabolism, glycolysis and gluconeogenesis, and propanoate and butanoate metabolism (Data Set S1).

10.1128/mBio.00575-18.9 DATA SET S1  Differential expression data and normalized patient read counts for reads pooled by EC number and for each ORF in the metatranscriptome. Download DATA SET S1, XLSX file, 35.9 MB. Copyright © 2018 Nowicki et al. 2018 Nowicki et al. This content is distributed under the terms of the Creative Commons Attribution 4.0 International license.

**FIG 5:**
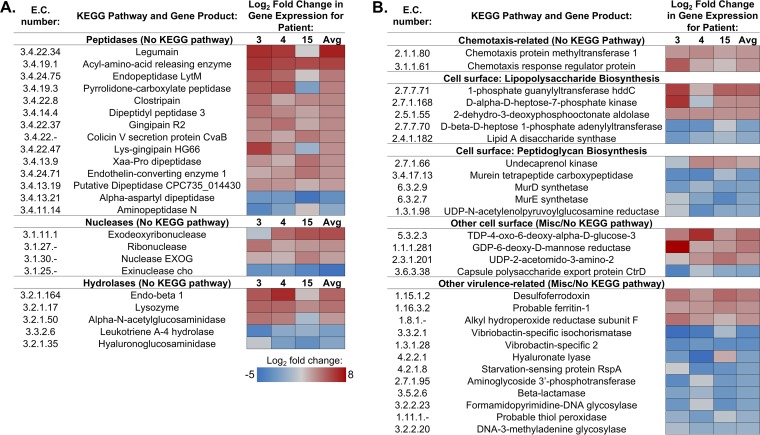
Virulence-related genes with significant differential expression between health and disease. Differential expression between samples collected from clinically diseased (MGI = 2, visit 6) and clinically healthy (MGI = 0, visit 3) teeth were compared and reported as significant if *P* was ≤0.05. Log_2_ fold change in gene expression was calculated for patients 3, 4, and 15 individually or pooled across all three patients. Shown here are hydrolytic enzymes (A) or other virulence-related genes (B) that are significantly up- or downregulated in samples from clinically diseased individuals relative to those from healthy patients.

We then directed our attention to genes with virulence-related activities with significant changes in expression in gingivitis relative to health. We defined virulence-related gene products as those involved in colonization of, enhanced survival within, or evasion of host or those that directly cause pathological damage associated with disease, according to the work of Wassenaar and Gaastra (28). In addition to products commonly associated with virulence such as adhesins and antibiotic resistance genes, additional gene products found during periodontal disease that meet these criteria include those involved in bone resorption and tissue destruction (29). Thirty of the 191 significantly upregulated EC enzymes had virulence-related functions (Fig. 5). Fold changes in expression for these genes within each patient and across all sequenced patients are shown in Fig. 5, with peptidases, nucleases, and hydrolases shown in Fig. 5A and those involved in chemotaxis, cell surface modifications, and other virulence activities shown in Fig. 5B.

The majority of nonspecific peptidases were upregulated during disease transition, supporting the idea that expression of these potentially destructive enzymes can promote periodontal disease (Fig. 5A). While some genes involved in the biosynthesis of cell surface features were significantly upregulated during gingivitis, others were significantly downregulated, including four genes involved in peptidoglycan biosynthesis. This suggests that changes to the cell surface or growth in general are dynamic during this early stage of disease. Genes involved in iron acquisition were similarly variably up- or downregulated in our data set. While *Vibrio*-specific siderophores were found to be downregulated during the early stages of periodontal disease, two other genes involved in iron acquisition were significantly upregulated across the entire metatranscriptome. Although the magnitude of gene expression changes varied among the three individuals sequenced as seen in other studies (14, 16), collectively our data suggest that changes in the overall activity of oral microbiota during the early stages of periodontal disease progression promote enhanced destruction of host tissue and survival within the oral cavity.

### Individual periodontal pathogens upregulate expression of both specific and general virulence-related genes during gingivitis relative to health.

Our next aim was to analyze the virulence-related activities of specific periodontal pathogens or opportunistic pathogens. We focused our analysis on representative species from the five most highly abundant genera present in our samples during disease (Fig. 4A): *Leptotrichia* (Leptotrichia buccalis), *Prevotella* (Prevotella nigrescens), *Streptococcus* (Streptococcus constellatus), *Fusobacterium* (Fusobacterium nucleatum), and *Actinomyces* (Actinomyces israelii). We first normalized the total read counts for each transcript in the metatranscriptome across the three patients analyzed (patients 3, 4, and 15), pooling samples collected from the same teeth during health (MGI = 0, visit 3) and during gingivitis (MGI = 2, visit 6) (Data Set S2). We then analyzed differential gene expression between health (samples collected from teeth with MGI = 0) and gingivitis (samples collected from teeth with MGI = 2). This gave an overview of differentially expressed genes within the entire microbial community during gingivitis relative to health (Data Set S2). We then looked at both known, characterized virulence-related genes associated with oral microbiota (collagenase, gingipain, and hemagglutinin) (16) and generalized virulence-related gene products known to promote bacterial colonization and survival in the oral cavity and to exacerbate periodontal disease (nonspecific peptidases or proteases and stress response proteins). Criteria for virulence traits selected were the same as described above for the overall metatranscriptome analysis; virulence-related genes with the highest fold changes in expression are shown. The differential expression data from the representative oral pathogens analyzed show a variety of virulence-related activities from these 5 organisms alone (Table 1).

10.1128/mBio.00575-18.10 DATA SET S2  Normalized patient read counts mapping to each ORF in the metatranscriptome. Download DATA SET S2, TXT file, 70.1 MB. Copyright © 2018 Nowicki et al. 2018 Nowicki et al. This content is distributed under the terms of the Creative Commons Attribution 4.0 International license.

**TABLE 1:** Upregulated virulence-related genes of representative periodontal pathogens from the five most abundant genera during gingivitis (MGI = 2)^a^

Organism	Locus tag	Gene product	Log_2_ fold change	*P* value
L. buccalis	lbuc_c_1_2002	DNA protection during starvation protein	8.4	0.000
	lbuc_c_1_1321	Response regulator MprA	8.3	0.000
	lbuc_c_1_356	Penicillin-binding protein 2	7.6	0.001
	lbuc_c_1_1097	Oligoendopeptidase F homolog	7.1	0.002
	lbuc_c_1_87	Toxin YoeB	6.6	0.005
	lbuc_c_1_416	Ferrous iron transport protein B	5.7	0.011
	lbuc_c_1_16	Multidrug export protein MepA	5.6	0.024
	lbuc_c_1_814	Extracellular serine protease	4.8	0.029
				
P. nigrescens	pnig_c_9_1227	Multidrug resistance ABC transporter	9.1	0.000
	pnig_c_5_880	Multidrug export protein MepA	8.8	0.000
	pnig_c_4_764	Superkiller protein 3	8.8	0.000
	pnig_c_14_1461	Multidrug resistance protein NorM	6.6	0.005
	pnig_c_16_1584	Endothelin-converting enzyme 1	6.2	0.001
	pnig_c_13_1445	Thiol protease/hemagglutinin PrtT	5.9	0.003
	pnig_c_18_1640	Collagenase	5.6	0.008
	pnig_c_3_568	Ferrous iron transport protein B	5.5	0.005
	pnig_c_3_496	Protease PrtH	5.4	0.007
	pnig_c_28_1930	Xaa-Pro dipeptidase	5.3	0.007
	pnig_c_10_1306	Putative surface protein BspA-like	5.3	0.011
	pnig_c_3_540	Gingipain R1	4.4	0.040
				
F. nucleatum	fnuc420_c_1_251	Cytosol nonspecific dipeptidase	8.0	0.000
	fnuc2539_c_1_1214	Oligoendopeptidase F homolog	7.8	0.001
	fnuc2539_c_1_2112	Exodeoxyribonuclease	7.6	0.001
	fnuc420_c_1_145	Thermostable carboxypeptidase 2	7.5	0.001
	fnuc420_c_6_1159	Ferric transport protein FbpB	7.4	0.002
	fnuc2539_c_1_1239	Iron import protein IrtA	7.0	0.004
	fnuc2539_c_1_1278	Multidrug resistance protein YoeA	6.9	0.004
	fnucp_c_3_1299	Penicillin-binding protein 1C	6.8	0.003
	fnuc2539_c_1_1432	Multidrug resistance protein NorM	6.7	0.006
	fnuc2539_c_1_924	Endoribonuclease YbeY	6.6	0.006
	fnuc2539_c_1_1998	Filamentous hemagglutinin	6.5	0.002
	fnuc2539_c_1_1429	Ferrous iron transport protein B	5.3	0.010
				
S. constellatus	scon_c_1_159	Alkyl hydroperoxide reductase subunit	5.8	0.018
	scon_c_3_1434	Regulatory protein Spx	5.7	0.019
	scon_c_2_864	Uncharacterized protease YdeA	4.9	0.047
	scon_c_2_968	Ferrous iron transport protein B	4.5	0.042
				
A. israelii	aisr_c_21_2065	Virulence-associated protein I	6.4	0.008
	aisr_c_31_2454	RNase VapC35	5.0	0.042
	aisr_c_11_1426	Putative DNase RhsC	3.9	0.047

^a^ Species analyzed include L. buccalis, P. nigrescens, S. constellatus, F. nucleatum, and A. israelii. Read counts were normalized for all ORFs within the metatranscriptome, and significantly differentially expressed genes pooled across three patients (3, 4, and 15) were determined by DESeq2. The log_2_ fold changes in gene expression shown here represent differential expression between samples collected from teeth with gingivitis (MGI = 2, visit 6) and from healthy teeth (MGI = 0, visit 3).

L. buccalis virulence-related gene products upregulated during gingivitis include several genes involved in antibiotic resistance and nonspecific proteases, as well as the response regulator MprA (Table 1). This gene has been shown in other organisms to play a role in regulation of genes crucial for pathogenesis (30) and thus may also play a role in L. buccalis pathogenicity. Both P. nigrescens and F. nucleatum significantly overexpressed a wide variety of virulence-related genes in plaque samples from teeth at a time point showing clinical indications of disease, including those involved in antibiotic resistance, proteolysis, breakdown of collagen, and iron uptake (Table 1). Of note, several gene products found to be increased in expression during gingivitis in our EC number analysis were found to be highly upregulated by P. nigrescens, including virulence factors endothelin-converting enzyme 1 and a gingipain. S. constellatus and A. israelii upregulated fewer genes across their entire genome and also fewer virulence-related genes than did the other three organisms analyzed (Table 1). In addition to upregulating genes involved in general survival mechanisms or nucleic acid degradation, however, these organisms also upregulated gene products specifically shown to be involved in virulence in other organisms: the Spx regulatory protein (31) (S. constellatus) and virulence-associated protein 1 (A. israelii) (32).

Upon analysis of the number of reads mapping to each of these species, we determined that upregulation of virulence-related genes in P. nigrescens was not necessarily induced, as an increase in both the number of active cells (as measured by rRNA sequences) and the transcript abundance of this species occurred in teeth experiencing gingivitis (MGI = 2) relative to the same teeth during health (MGI = 0) (Fig. S5B and C). Similarly, L. buccalis 16S rRNA v4-v5 amplicon levels were also significantly elevated during gingivitis (Fig. S5B), although transcript abundances of this species were not significantly different between the two disease states (Fig. S5C). Despite the fact that increased expression of virulence-related genes is possibly due to an overall increase in the abundance of P. nigrescens and L. buccalis, it is still worth noting the changes in the overall functional repertoire of the oral metatranscriptome that can potentially promote periodontal disease progression; thus, we have included these genes in Table 1. On the other hand, relative 16S rRNA v4-v5 amplicon or transcript abundance of S. constellatus, F. nucleatum, and A. israelii did not significantly increase in samples collected from teeth with gingivitis relative to health (Fig. S5B and C), suggesting that virulence-related gene products of these organisms are upregulated in their expression levels during disease as opposed to being elevated as a result of increased species abundance.

## DISCUSSION

In our analysis of dental plaque samples during the transition from health to gingivitis, we began by first analyzing community composition through a beta-diversity analysis. Composition and diversity of our samples were found to be significantly correlated with clinical index of disease severity (MGI score) (Fig. 2B). Samples collected from teeth with no visible evidence of periodontal disease (MGI = 0) cluster distinctly from those originating from teeth with the highest clinical disease score in our study (MGI = 2). The clustering of our metatranscriptome sequencing data by MGI score (Fig. 4B) along with our identification of differentially expressed virulence-related genes between samples from teeth with an MGI score of 0 and samples from teeth with a score of 2 lends support to the efficacy of MGI score as an indicator of disease progression. We also found that samples originating from the same patient were statistically more similar in composition than those from different patients (Fig. 2C), although samples still clustered by MGI score despite this patient effect. Our data do suggest that certain patients have more stable microbiota community structure while others were subject to greater community changes over the time course in which this study took place (Fig. 2C). This implies that patient-specific factors not considered in this analysis, such as host genetics, comorbidities, age, or sex, could play an important role in shaping oral microbiota community structure.

Surprisingly, our beta-diversity analysis revealed that the oral hygiene status (i.e., whether or not teeth were brushed) had no significant impact on microbial community composition (Fig. 2A). This is in contrast to at least one previously published study that found a significant decrease in plaque (as measured by gingival index) after brushing (8). These patients, however, were subjected to a rigorous oral hygiene regimen to obtain this decrease. As it was not possible to ensure patient compliance with all study parameters during the course of the experimental gingivitis study, it is plausible that some patients did not adhere to the prescribed oral hygiene regimen. In agreement with this, Kistler and colleagues have published that many individuals do not self-apply oral hygiene techniques that are sufficient to prevent the onset and progression of gingivitis (9). Although we were unable to accurately quantify the total raw number of bacteria present at any given time point (data not shown), it is also possible that brushing can affect the total number of microbes present rather than the diversity and relative abundances of different taxa. The net changes in levels of three different proinflammatory mediators (IL-8, MMP-8, and MMP-9) measured in the gingival crevicular fluid (GCF) of sampled teeth also had no significant contribution to microbial community composition (see Fig. S2B, C, and D in the supplemental material). As one report found that the levels of most inflammatory cytokines vary significantly between individuals (33), quantification of these particular mediators may not be a reliable predictor of subgingival plaque community structure.

Our analysis of relative genus abundance within the subgingival plaque communities through both 16S rRNA sequencing and metatranscriptome sequencing revealed significant shifts during disease transition, although the two methods showed differences in the specific genera found to be most abundant (Fig. 3B and 4A). For example, while both 16S rRNA and metatranscriptome sequencing revealed *Streptococcus* and *Neisseria* to have higher abundance in samples collected from teeth at a point of clinical health, *Prevotella* was reported as a highly abundant genus during gingivitis based on metatranscriptome sequencing only (Fig. 4A). This finding underscores the limitations of either method in determining genus abundance with total accuracy. Despite these differences, rank abundance analysis and ratios of composition (16S rRNA data) to activity (RNA-seq data) of genera show similar trends in the two data sets (Fig. 4C and S6). In addition to analyzing relative abundance in our plaque samples, we also analyzed fold changes in genera (Fig. S5A). The most discordant genera in total abundance between samples collected from teeth during health and gingivitis are not necessarily the highest in abundance (Fig. 4A) according to our metatranscriptome data. While it would be interesting to further analyze specific functional contributions of these highly discordant genera during disease relative to health, limitations in total reads obtained made this difficult or impossible. For example, the lack of reads obtained for the genus *Tannerella* during health (Data Set S2) made further analysis of differential gene expression within this genus impractical, as virtually all genes show an increase in expression.

When analyzing our metatranscriptome data, we first wanted to get an overall idea of the general functional properties of the plaque communities during the transition from health to gingivitis. We thus began by pooling gene products (by EC number) across all taxa and found that 8.5% of pooled gene products were significantly upregulated during gingivitis, while 8.0% were significantly downregulated (Data Set S1). Genes involved in ascorbate and aldarate metabolism were upregulated during health, as seen in several previous studies (34, 35). Other metabolic pathways more strongly associated with health: carbon fixation in prokaryotes, the pentose phosphate pathway, and pyruvate metabolism. Interestingly, porphyrin and chlorophyll metabolic gene products were also found to be associated with health. Genes involved in metabolic pathways, including vitamin B_6_ metabolism and glycolysis and gluconeogenesis, were found in our data analysis. As seen in other studies, metabolic pathways more strongly associated with disease were pyrimidine metabolism (17) and propanoate and butanoate metabolism (16, 22, 36).

We then focused our attention on significantly differentially expressed genes with virulence-related activity (Fig. 5). These enzymes were involved in a variety of activities, including hydrolysis or proteolysis, degradation of nucleotides, chemotaxis, synthesis of cell surface structures (adhesion, motility, and protection), and a variety of other virulence functions (Fig. 5). Increased proteolytic and nucleolytic activities as seen in our data likely lead to the destruction of gum tissue and disturbance of the host immune system characteristic of periodontal disease and have been noted as potential drivers of periodontal disease in other recent metatranscriptome studies (15, 21, 22). Two different gingipains, or proteases that have previously been associated with periodontal disease in several studies (16, 37–39), were significantly upregulated in our data. While general proteolytic, nucleolytic, and several chemotaxis-related gene products showed increased expression, a number of other virulence-related gene products were downregulated in our data, suggesting the variable and unstable state of the microbiome during the early stages of gingivitis onset. Interestingly, several virulence-related gene products downregulated during disease relative to health may play a role in exacerbating the diseased state. For example, leukotriene A4 hydrolase is typically involved in alleviating inflammation (40); thus, downregulation of this gene could lead to the promotion of an inflammatory state typical of periodontal disease. Downregulation of starvation-sensing protein RspA leads to an increase in the production of metabolites that signal starvation (41) and thus can lead to increased survival during nutrient limitation.

Finally, we wanted to analyze the contributions of specific pathogens to virulence. We chose to focus on representative pathogens or opportunistic pathogens from the 5 most abundant genera during clinical disease (MGI = 2) (Fig. 4A): P. nigrescens from *Prevotella*, F. nucleatum from *Fusobacterium*, L. buccalis from *Leptotrichia*, S. constellatus from *Streptococcus*, and A. israelii from *Actinomyces*. P. nigrescens and F. nucleatum were selected as representative pathogens because of their frequent presence in periodontal infections (42, 43). Other oral metatranscriptome studies have also shown that both of these organisms increase expression of virulence factors during periodontal disease (21, 22). From the genus *Leptotrichia*, we chose to look at L. buccalis as it is the best-studied species of the genus and the primary causative agent of opportunistic *Leptotrichia* oral infections (44, 45). Although many members of the *Streptococcus* genus are commonly occurring commensals, S. constellatus is an opportunistic periodontal pathogen that is well documented in periodontal disease and thus is our choice for a representative of the genus *Streptococcus*. Along with other oral streptococci, S. constellatus can also be synergistically pathogenic with Porphyromonas gingivalis (42, 43). Finally, although most members of the genus *Actinomyces* are associated with oral health, we selected Actinomyces israelii for further analysis because it is the causative agent of a rare but severe infection, actinomycosis (46).

Within these 5 species, L. buccalis, P. nigrescens, and F. nucleatum overexpressed a variety of virulence-related genes and thus likely actively contributed toward disease progression, while S. constellatus and A. israelii overexpressed a more limited number of virulence genes during gingivitis (Table 1). Of note, P. nigrescens overexpressed a gingipain and several arginine/lysine-specific proteases during gingivitis (Table 1). In addition to degrading hemoglobin, these enzymes are able to degrade host cytokines and thus dampen the immune response to pathogens (47, 48). Interestingly, the metalloendopeptidase endothelin-converting enzyme was significantly overexpressed by P. nigrescens during gingivitis (Table 1). This enzyme can serve as a potent vasoconstrictor during cardiovascular disease (49), a common comorbidity that can result following periodontal disease (50). Other gene products with more generalized virulence-related activities that increased in expression among these organisms included peptidases, proteases, nucleases, and iron uptake proteins, gene products well known to be involved in virulence in all organisms.

This study is unique in that we have assessed changes in both subgingival microbiota community structure and function associated with the transition from health to periodontal disease. Our findings regarding both the shifts in community structure and functional changes seen between health and gingivitis are largely corroborated by similar findings in previous work analyzing samples from healthy and diseased (periodontitis) teeth. These data therefore characterize how microbial activities change during the early stages of periodontal disease. Our hope is that the virulence-related genes identified here will serve as candidates in future studies for prophylactic targets that can potentially prevent progression from gingivitis to more severe forms of periodontal disease.

## MATERIALS AND METHODS

### Patient population.

Samples were analyzed from a total of 10 patients who completed the present study. All patients were consulted and treated in the Graduate Periodontics Clinic at the University of Louisville School of Dentistry. The study was conducted in compliance with modern ethical guidelines, as approved by the local ethics board (study no. 14.0230). The study protocol was explained both verbally and in writing, and written informed consent was obtained from each participant prior to dental examination and sampling. The following were inclusion criteria in order for a patient to participate: (i) at least 18 years of age and good general and oral health, (ii) at least 20 natural teeth present, and (iii) baseline mean gingival index of less than or equal to 1 (according to the scoring by the modified gingival index [MGI]). Conditions that would exclude an individual from participating were (i) history of conditions requiring prophylactic antibiotic coverage prior to this study; (ii) use of antibiotic, anti-inflammatory, or anticoagulant medication within 1 month prior to the study; (iii) history of tobacco use; (iv) participation in another oral study involving oral care products concurrent or within 30 days of beginning this study; (v) pregnancy or lactation; (vi) significant oral tissue pathology (excluding gingivitis); (vii) moderate to advanced chronic periodontitis or other form of periodontal disease; and (viii) an underlying genetic or immunologic condition that might influence the study (e.g., diabetes or immunodeficiency).

### Study design.

Patients who met all inclusion criteria during the first visit underwent a thorough periodontal examination by a trained dental hygienist. A professional dental cleaning was conducted, and instructions for proper oral hygiene were provided. Participants returned for a second visit (visit 2) within 2 weeks of the initial visit, at which time the study began. At visit 2, baseline clinical data were recorded, and both plaque and gingival crevicular fluid (GCF) samples were collected from the first molars. If the first molar was missing, samples were collected from the second molar. When patients brushed and flossed during the study, an acrylic stent was worn on either the top or bottom arch of teeth; these teeth were considered unbrushed (Fig. 1). Whether the stent was worn on the top or bottom was randomly decided for each participant; half of the patients wore the stent on the top arch while half wore the stent on the bottom arch. The function of the stent was to prevent “unbrushed” teeth from being cleaned during the study, and thus the stent was worn only during brushing and flossing. Patients were instructed not to use any other oral care products (such as interdental cleaning aids, mouthwash, or chewing gum) during the course of the study. After the baseline visit, subgingival plaque and GCF were collected two more times, at 3 days after visit 2 (visit 3) and up to ~3 weeks after visit 2 (visit 6). The day of the third sampling was determined by the amount of gingivitis progression; patients continued with the study until their MGI score was at or above 2.

### Clinical assessment of gingivitis.

In addition to collecting plaque samples, calibrated examiners performed periodontal evaluations on the study participants. Examiners were calibrated to a gold standard examiner for probing depth (PD), plaque index (PI), and gingival index (GI). The examiners were calibrated until the agreement coefficient (kappa statistic) was at least 0.90 for the PD, PI, and GI with the gold standard examiner. Calibrated examiners scored patient teeth for experimental gingivitis using the modified gingival index (MGI) on both the buccal and lingual marginal gingiva. Scores were given according to the following criteria: 0 = normal (absence of inflammation), 1 = mild inflammation of a single portion of the gingival unit (characterized by a slight change in color but little change in texture), 2 = mild inflammation of the entire gingival unit, 3 = moderate inflammation (moderate glazing, redness, edema, and/or hypertrophy) of the gingival unit, and 4 = severe inflammation (marked redness and edema/hypertrophy, spontaneous bleeding, and ulceration of the gingival unit) (Fig. 1). Probing depths were measured (in millimeters) at a total of six sites per tooth on both the buccal and lingual marginal gingiva, and average measurements were recorded.

### Subgingival plaque and gingival crevicular fluid sampling.

GCF samples were collected from 8 sites by inserting paper strips into the sulcus of each site for a total of 30 s. GCF volume was measured using an electronic measuring device. Subgingival plaque samples from two brushed or two unbrushed teeth from each individual were collected at the three time points after the onset of the experimental gingivitis study. The two brushed or unbrushed plaque samples at each time point were pooled prior to nucleic acid extraction and then immediately frozen at −20°C until further sample processing. Subgingival plaque samples were collected from the same sites during the course of the study by inserting one sterile endodontic paper point (Dentsply Caulk, Milford, DE) into the sulcus of each tooth for 10 s, followed by scraping with a curette. Upon collection of subgingival plaque, samples were immediately placed into a microcentrifuge tube containing RNAlater (Invitrogen) and stored at −20°C until further sample processing. Samples from two brushed or two unbrushed teeth were pooled to ensure that enough microbial cells were collected in order to have sufficient amounts of DNA and RNA for downstream analyses and also to account for the inherent differences seen from tooth to tooth within the same individual.

### Cytokine and matrix metalloproteinase analysis.

Paper strips used to collect GCF were thawed on ice, and GCF was eluted by adding 200 µl elution buffer (50 nM Tris-HCl, 5 mM CaCl_2_, 0.2 M NaCl [pH 7.6], 1 mg/liter antipain, 1 mg/liter aprotinin, 125 mg *N-*ethylmaleimide, and 50 mg detergent [Zwittergent 3-12; EMD Millipore]). Elution was achieved by vigorous vortexing for 15-min intervals for a total of 1 h. Inflammatory mediator analysis was performed using a commercially available multiplexed bead-based assay designed to quantitate multiple analytes. IL-8, MMP-8, and MMP-9 were measured using Luminex technology in pooled GCF samples.

### Total RNA isolation.

Total RNA was prepared from frozen subgingival plaque samples stored in RNAlater (Invitrogen) as described previously (16). One-half of the purified RNA was used for rRNA sequencing of the v4-v5 region of 16S rRNA cDNA, while the other half of the sample was saved for subsequent treatment with Ribo-Zero and RNA-seq library preparation.

### rRNA sequencing.

rRNA sequencing of the v4-v5 region of 16S rRNA was performed as previously described by Jorth et al. (16). Briefly, total RNA was reverse transcribed into cDNA using the random primer 16S926RT (Table S2). The ~500-bp v4-v5 region of the 16S rRNA gene was then PCR amplified from cDNA using primers 16SV4515F and one of the 24 uniquely bar-coded reverse primers (Table S2). Custom primers were then used for sequencing on the Illumina MiSeq platform (Table S2).

10.1128/mBio.00575-18.8 TABLE S2  Primers used in this study. Download TABLE S2, TIF file, 2.6 MB. Copyright © 2018 Nowicki et al. 2018 Nowicki et al. This content is distributed under the terms of the Creative Commons Attribution 4.0 International license.

### Oral microbiota community analyses.

The 16S rRNA v4-v5 MiSeq sequencing reads were assembled, mapped, and analyzed using Python scripts within the open-source phylogenetic DNA analysis pipeline QIIME (51). Paired-end reads were assembled and then quality filtered using the QIIME Python scripts multiple_join_paired_ends.py and multiple_split_libraries_fastq.py. Reads were mapped to the Human Oral Microbiome Database (HOMD) 16S rRNA reference (v14.51) to determine the number of reads mapping to each distinct OTU using the QIIME Python script pick_closed_reference_otus.py. Relative abundances of each OTU were then summarized and visualized using the QIIME Python scripts summarize_taxa.py and summarize_taxa_through_plots.py. Sequencing reads were rarefied 10 times at each step with a step size of 500 sequences from 500 to 5,000 sequences using the QIIME Python script multiple_rarefactions.py. Mean alpha-diversity (within-sample diversity) was calculated using the QIIME Python scripts alpha_diversity.py and collate_alpha.py. These scripts were used to determine the Shannon index for each sample or group of samples, which is a measure of species richness within samples. Beta-diversity (between-sample) analysis was performed by running the QIIME Python script beta_diversity.py using Bray-Curtis dissimilarity analysis as a measure of beta-diversity. Samples were then clustered by principal-coordinate analysis using the QIIME Python script principal_coordinates.py. Samples were categorized, and significant differences between categories were determined by PERMANOVA statistical analysis using the QIIME Python script compare_categories.py. For analysis of relative genus abundance in samples, OTUs not present in at least 2 samples or present at less than 1% abundance in any sample were filtered from the data using the QIIME Python script filter_otus_from_otu_table.py. Relative abundance data were then calculated using the summarize_taxa_through_plots.py script. To calculate genera with significant changes in abundance between sample groups, relative abundances were first converted to absolute read counts using the summarize_taxa.py script with the –a flag. The QIIME Python script group_significance.py was the used to calculate significant changes, using the Kruskal-Wallis analysis as the significance test.

### RNA-seq library preparation.

Prokaryotic and eukaryotic rRNAs were removed from total RNA using the Ribo-Zero epidemiology kit (Epicentre). mRNA was purified, fragmented, and precipitated as described by Jorth et al. (16). RNA-seq libraries were constructed using the NEB Next Multiplex small RNA library preparation set for Illumina according to the manufacturer’s protocol. The cDNA libraries obtained at the end of this prep were stained using SYBR gold nucleic acid stain (Invitrogen) and visualized using a Gbox imaging system. cDNA between ~150 and 300 bp was extracted according to the NEB quality control (QC) check and size selection protocol (protocol E7300) and resuspended in RNase-free water. Library cDNA concentration was determined using a Qubit fluorometer (Thermo Fisher), and size distribution was analyzed on an Agilent Bioanalyzer. Single-end 50-bp sequencing was performed at the University of Texas Genomic Sequencing and Analysis Facility (UT GSAF) on an Illumina HiSeq2000 system.

### RNA-seq fastq read processing.

HiSeq reads were downloaded and concatenated, and adapters were removed using Flexbar (v2.34) (52), as described previously (16). Reads were trimmed to a minimum length of 18 bp or 30 bp, depending on the analysis. An absolute minimum length of 18 bp was selected for the pooled reads to minimize the likelihood of random, nonspecific mapping to our reference metagenome. Since these functional data are pooled across all species by EC number and did not aim to detect specific species-level reads, this amount of random mapping would affect our results only minimally (Fig. S4B). For species-specific functional analysis, 30-bp trimmed reads were used to obtain a minimum of 10× coverage of the reference metagenome (Fig. S4A). For samples lacking enough 30-bp trimmed reads to obtain 10× coverage, reads between 18 and 30 bp were used to ensure adequate coverage of the search space.

### Reference metagenome assembly.

The reference metagenome was generated as previously described (16). All annotated human oral microbiome genome sequences and annotations were downloaded in both Fasta and GFF formats from the open-access, online Human Oral Microbiome Database (HOMD) (53). Genome sequences were downloaded, concatenated, and processed to remove non-protein-coding genes using the Perl scripts GenomeMerge.pl and HOMDpull.sh to create an annotated metagenome to which to map our sequencing reads. If an EC number was associated with a gene, it was downloaded from KEGG (54) using the custom Perl scripts PullEC.pl and HOMD_GenomeMerge.pl. Custom scripts are available at the following web address: http://github.com/khturner/metaRNA-seq.

### RNA-seq read mapping and normalization.

Trimmed RNA-seq reads were mapped against the reference metagenome using a Bowtie 2 end-to-end alignment, very-sensitive parameters (55). The species read match with the highest MAPQ score for each read was kept; other potential matches with lower quality scores were discarded. In the event of multiple matches with equal quality scores, Bowtie 2 selected the match to be kept at random (55). After mapping with Bowtie 2, reads with >2 mismatches and unmapped reads were discarded using the UNIX grep command. To compare samples across the same condition, within-sample normalization was performed by dividing the counts to each genus by the highest-activity genus for that sample. The statistical power of our study, and thus the likelihood of identifying a true positive, was found to be 0.8 when the effect size was set at 2.75-fold cutoff, calculated using the R program RNASeqPower (v3.5) (56) within the Bioconductor package using a sample size of 3, a false-discovery rate of 0.05, and the lowest sequencing depth in all libraries (determined through SAMtools mpileup).

### Differential expression analysis.

After removing unmapped or highly mismatched reads and sorting the remaining reads, the number of reads mapping to each ORF was determined using the open-source Python library HTSeq (57). To analyze reads corresponding to specific species-level ORFs, genus, species, and product data were overlaid with the count matrix resulting from HTSeq using UNIX commands (Data Set S2). To analyze reads pooled across all organisms by EC number, EC numbers corresponding to ORFs were overlaid with the count matrix, and genes without an EC number were removed from the list using UNIX commands. Counts for ORFs from different species but corresponding to the same EC number were then combined using the data.groupby command within the open-source Python library pandas (Data Set S1). For both species-specific ORF read counts and pooled EC number counts, count tables were imported into RStudio. Differential expression was then assessed using the open-source R package DESeq2 (27). Low- versus high-MGI score samples were analyzed in a paired manner, to account for differences between patients. Differential gene expression across all patients was analyzed by grouping the individual patient read counts into MGI = 0 or MGI = 2 categories in DESeq2, treating each patient as a replicate in each category. Differential gene expression for individual patients was calculated from patient raw read counts normalized using DESeq2; read counts for specific EC numbers analyzed obtained for MGI = 2 samples were divided by counts for MGI = 0 samples. For samples with <1 read for a particular gene, a minimum read count of 1 was used in order to calculate fold changes.

### Accession number(s).

All samples have been uploaded to the NCBI Sequence Read Archive (SRA) under BioProject ID PRJNA387475.
