# Supplementary figures and images for: Microbiota and Metatranscriptome Changes Accompanying the Onset of Gingivitis
Source: mBio. 2018 Apr 17;9(2):e00575-18. doi: 10.1128/mBio.00575-18 (PMC5904416; doi:10.1128/mBio.00575-18)

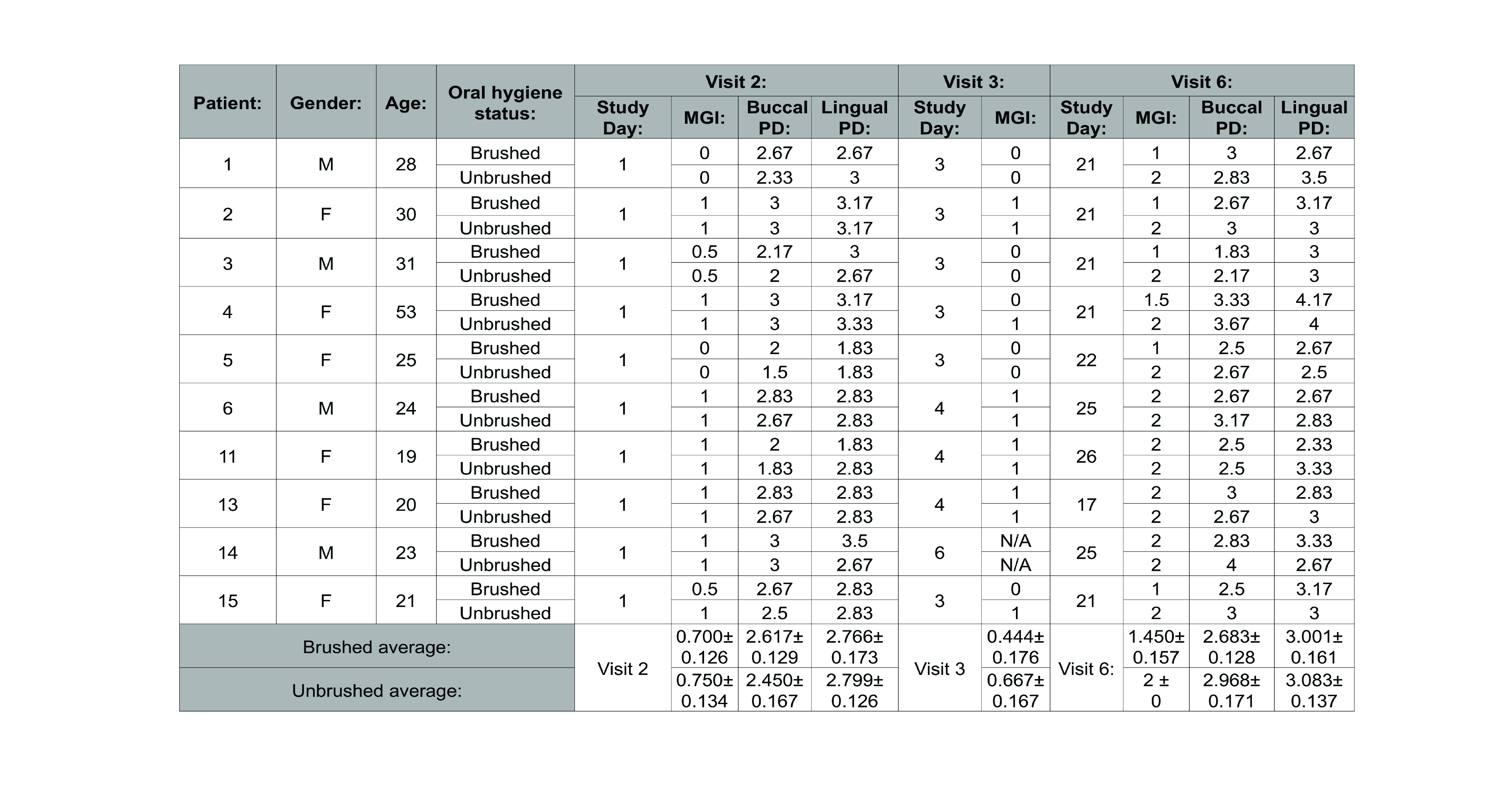

Supplement: TABLE S1 [file mbo002183835st1.tif]

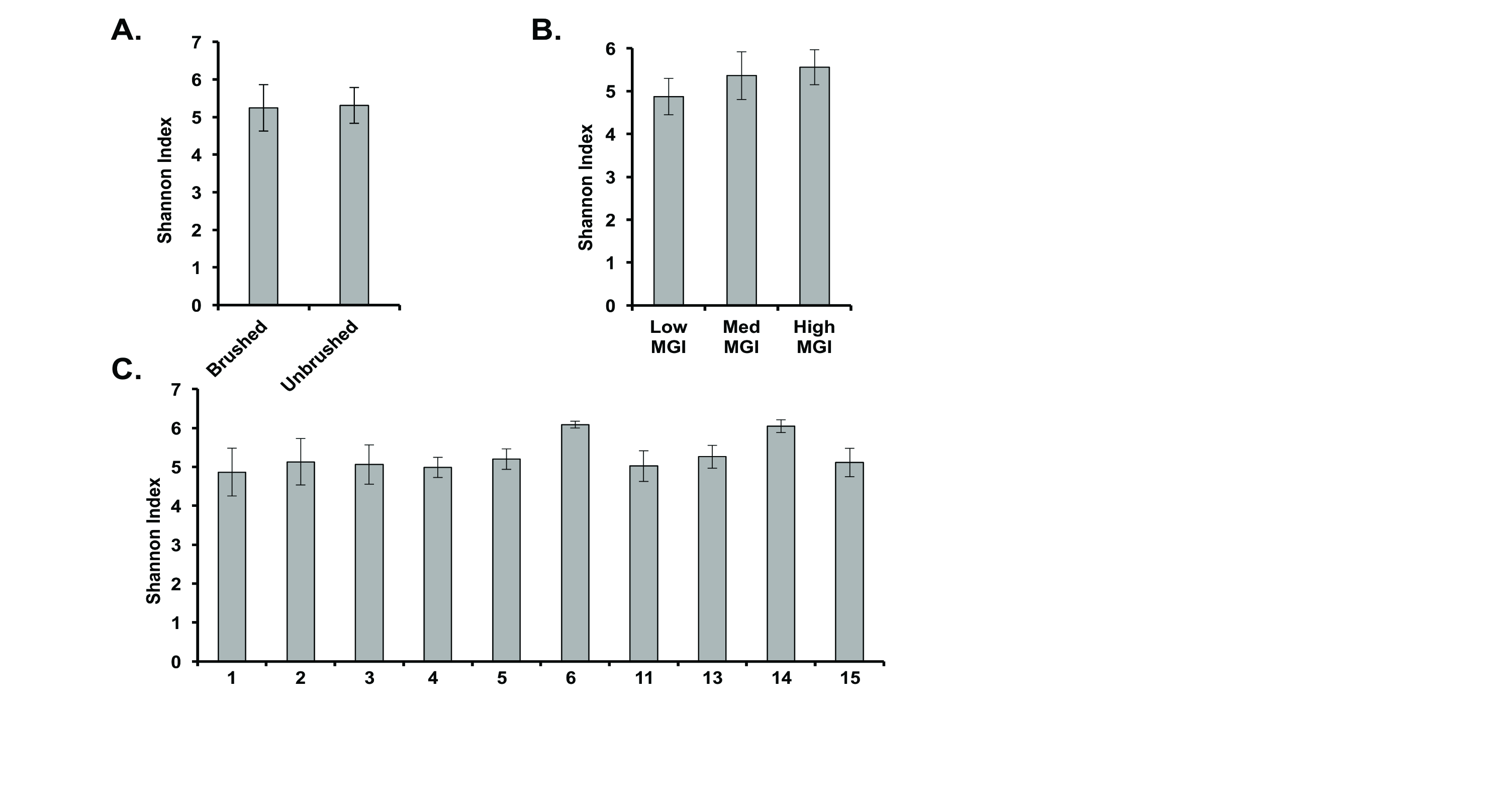

Supplement: FIG S1 [file mbo002183835sf1.tif]

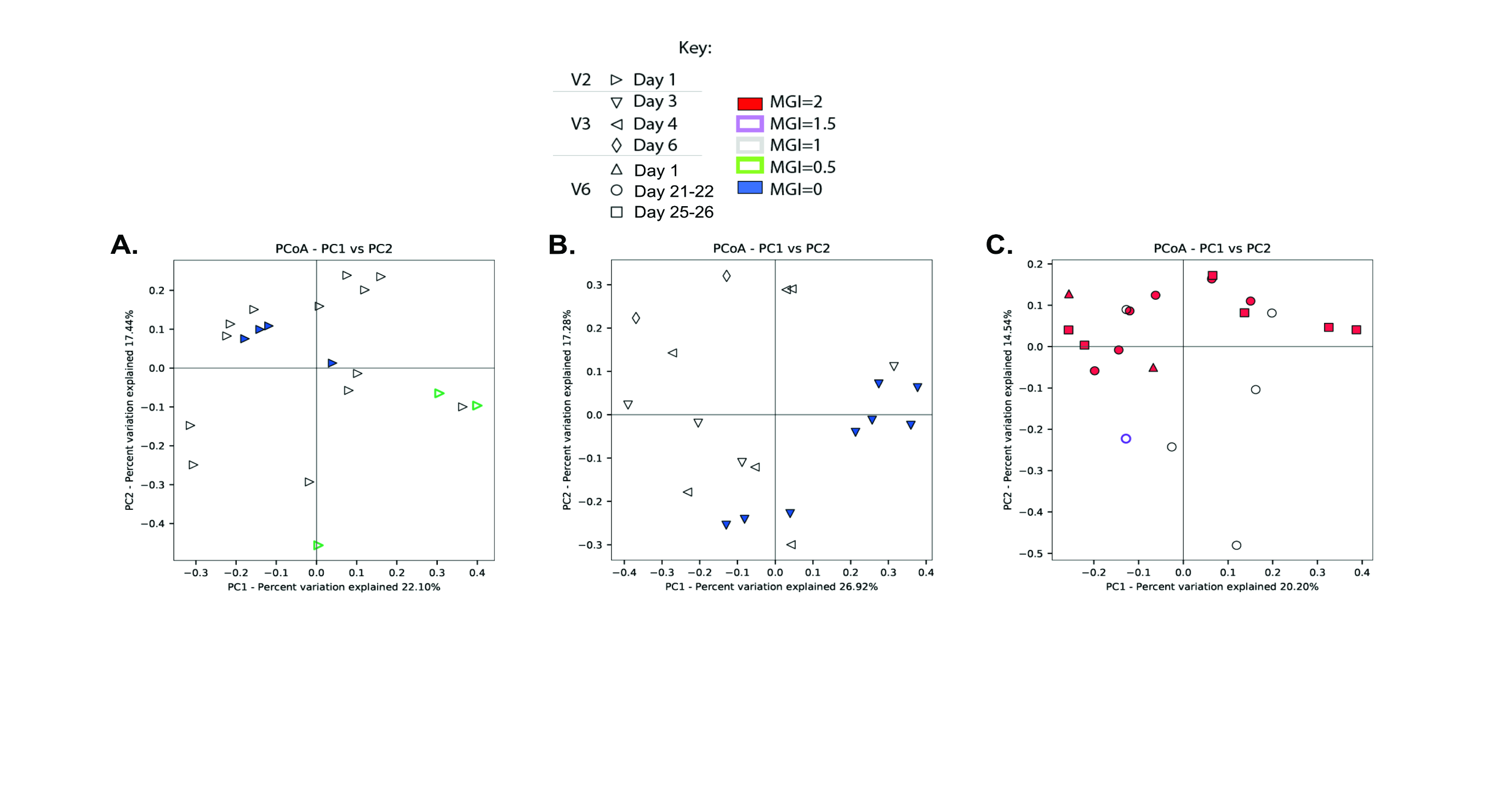

Supplement: FIG S2 [file mbo002183835sf2.tif]

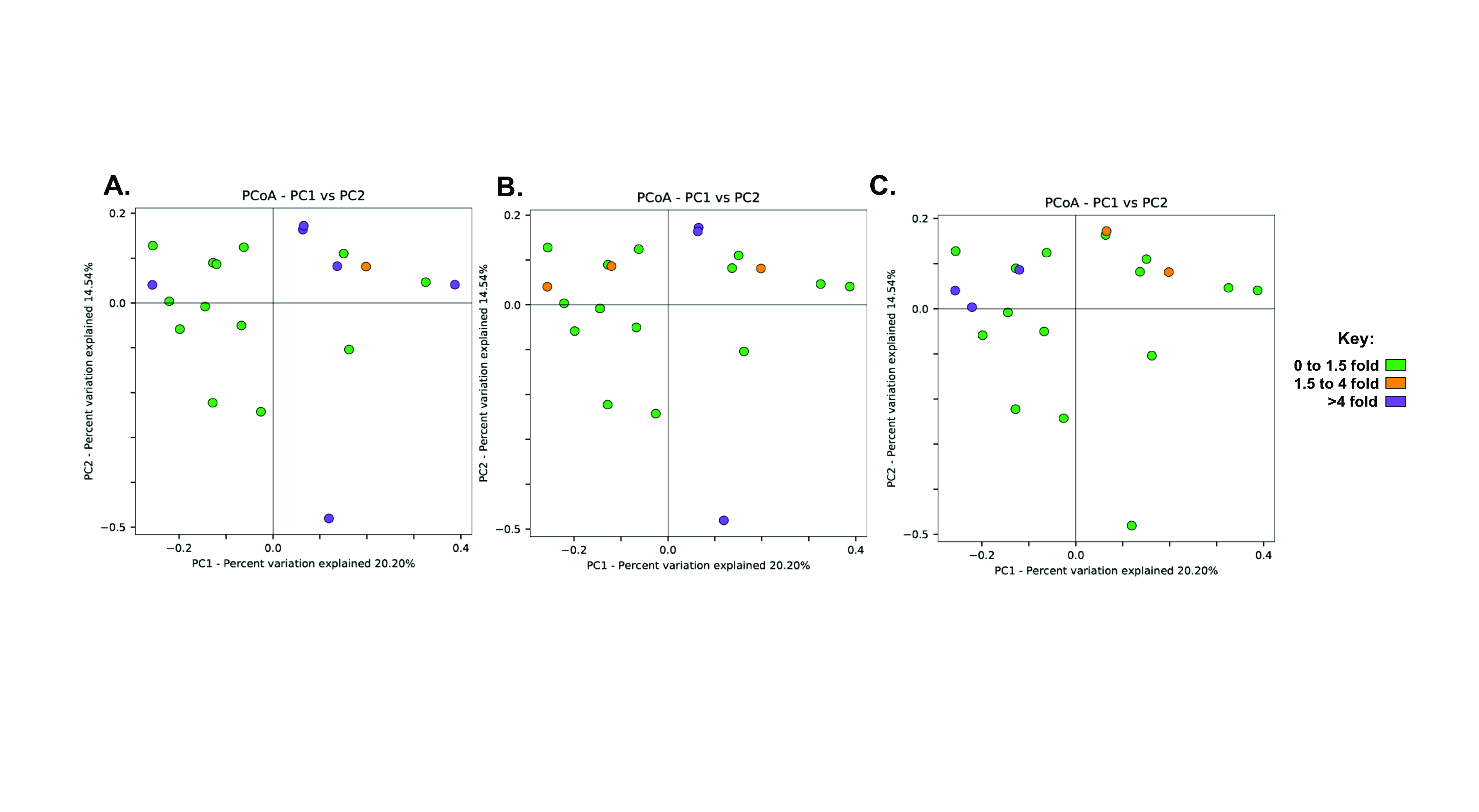

Supplement: FIG S3 [file mbo002183835sf3.tif]

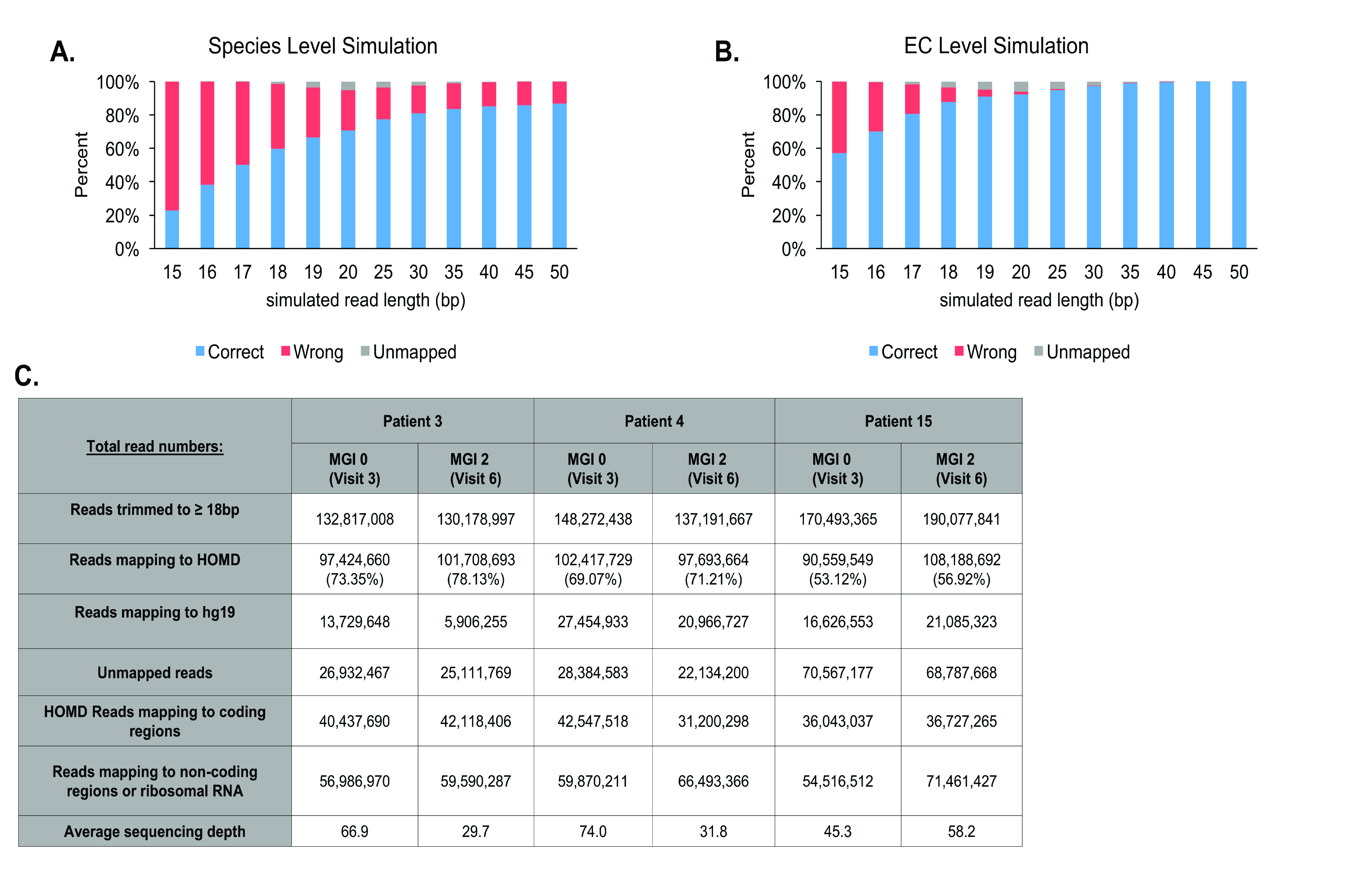

Supplement: FIG S4 [file mbo002183835sf4.tif]

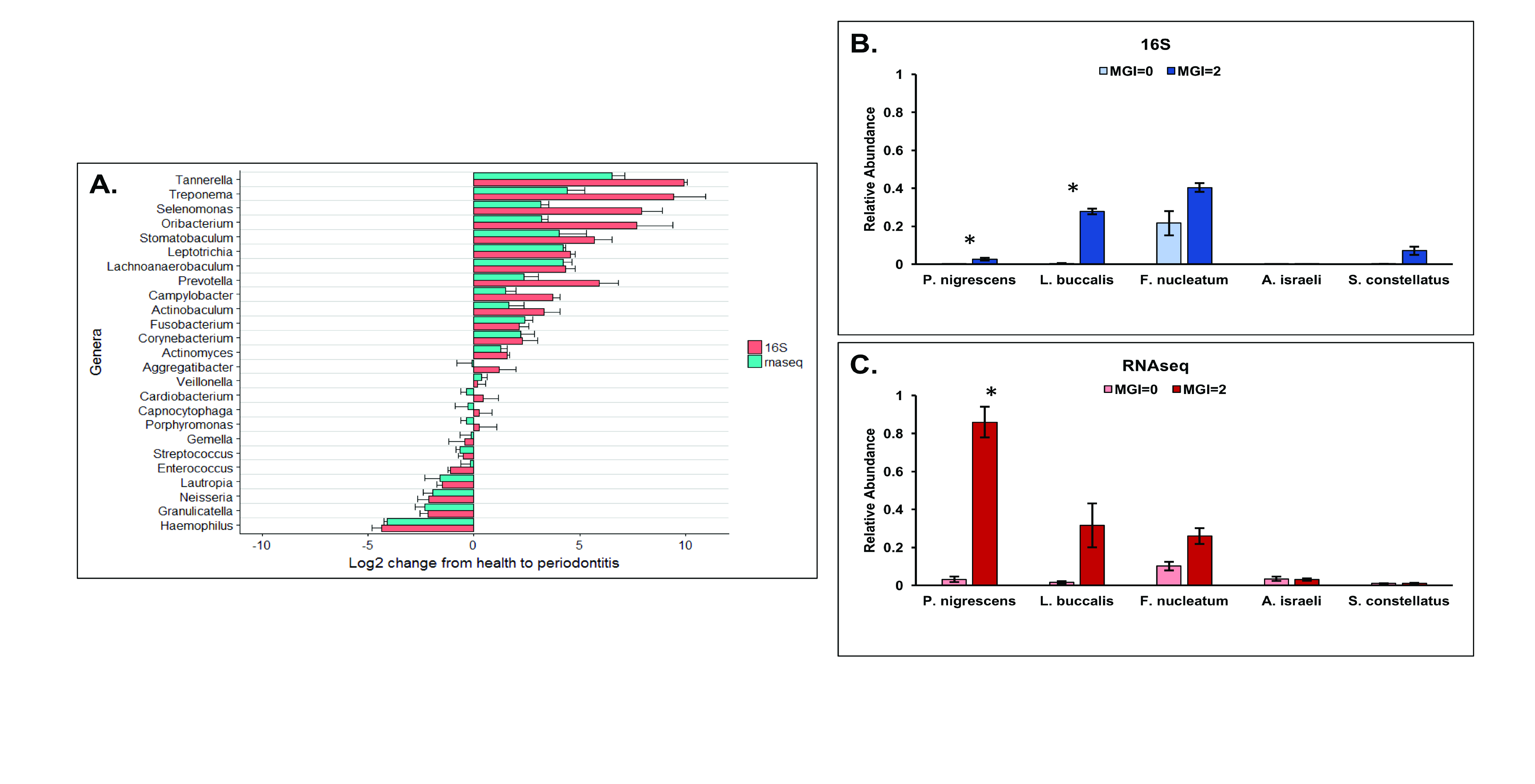

Supplement: FIG S5 [file mbo002183835sf5.tif]

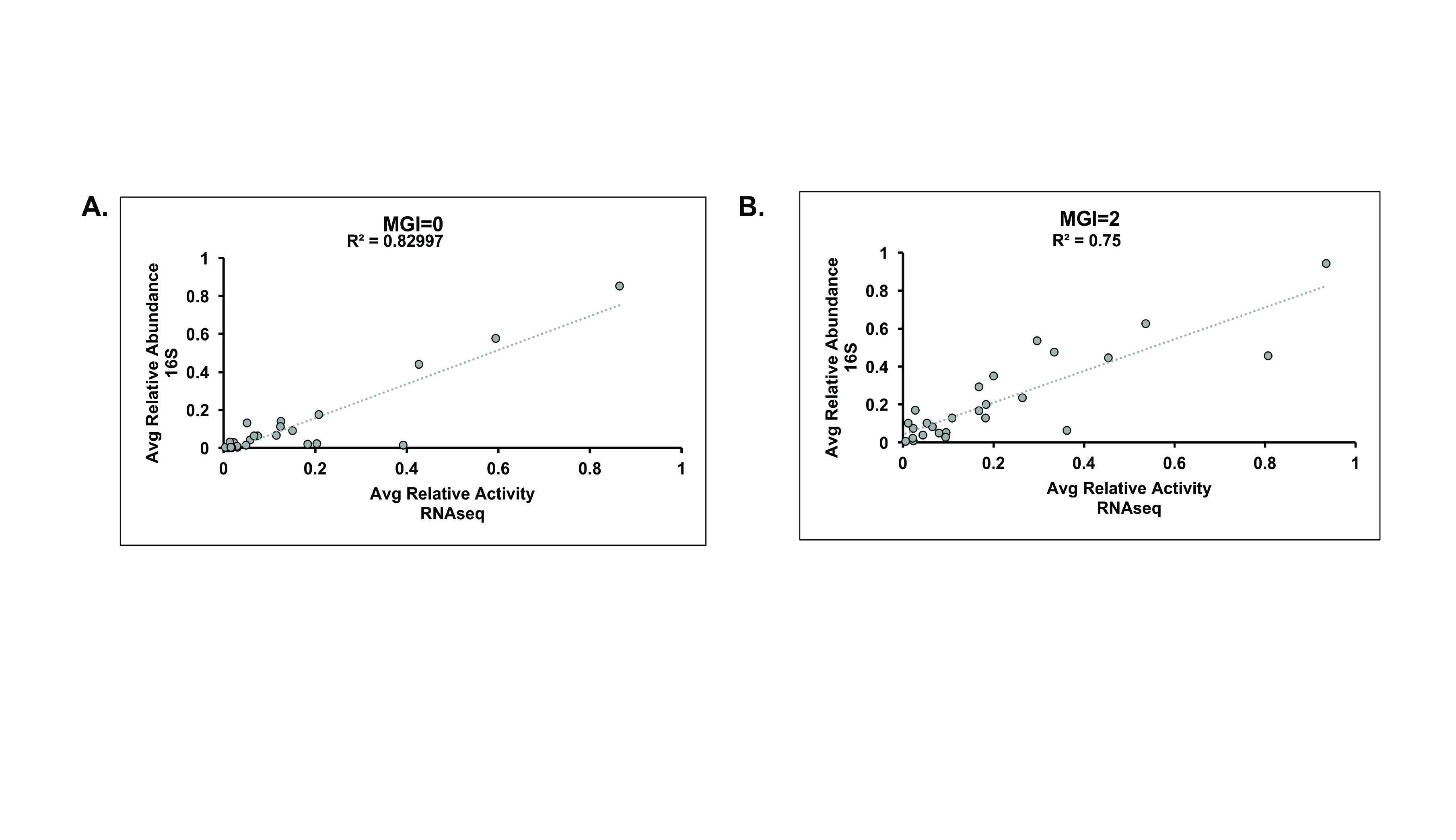

Supplement: FIG S6 [file mbo002183835sf6.tif]

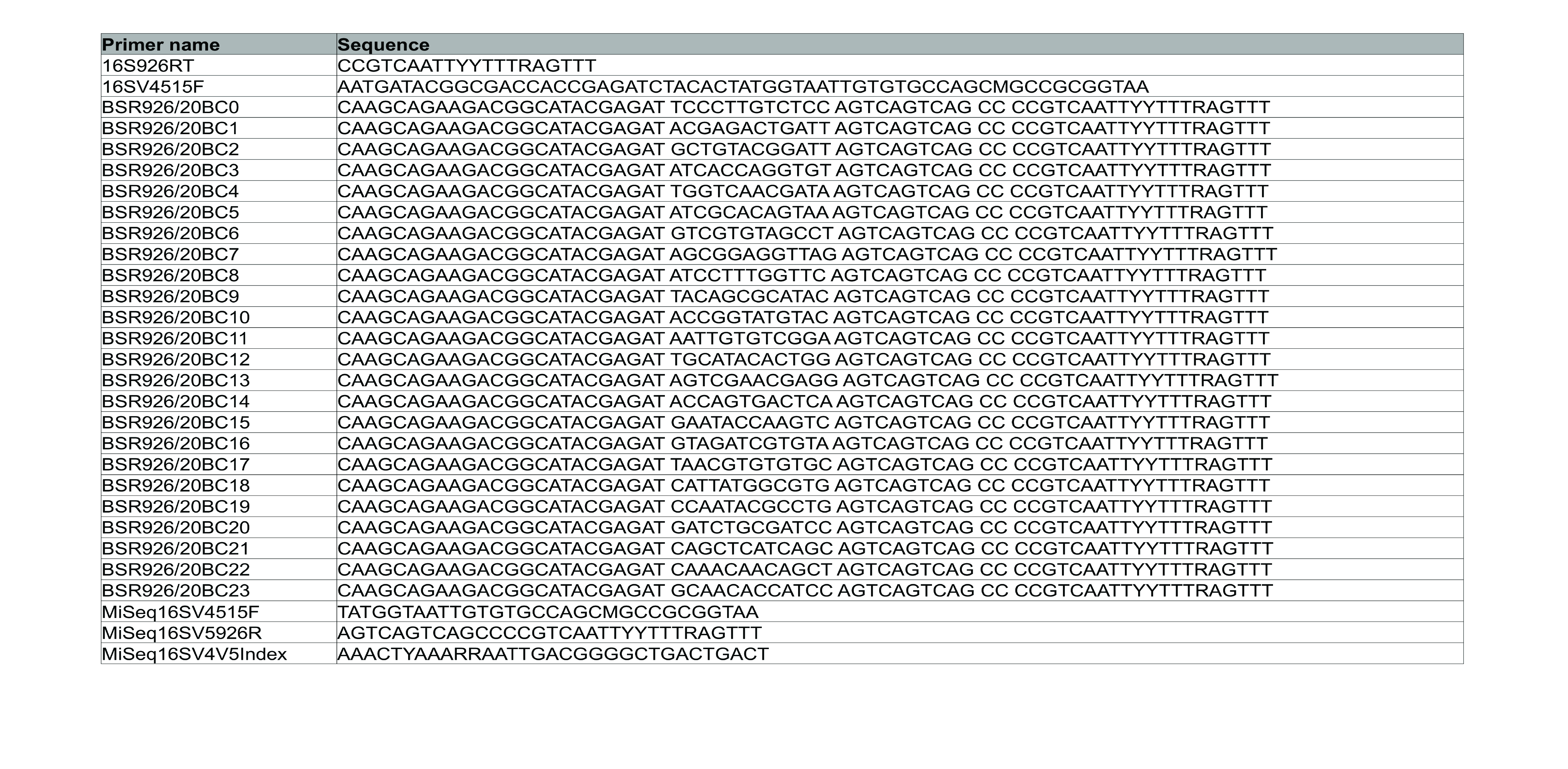

Supplement: TABLE S2 [file mbo002183835st2.tif]
